# GII.P16-GII.2 Recombinant Norovirus VLPs Polarize Macrophages Into the M1 Phenotype for Th1 Immune Responses

**DOI:** 10.3389/fimmu.2021.781718

**Published:** 2021-11-18

**Authors:** Ji Cheng Han, Qiu Xuan Li, Jin Bo Fang, Jin Yong Zhang, Yi Quan Li, Shan Zhi Li, Cheng Cheng, Chang Zhan Xie, Fu Long Nan, He Zhang, Zhuo Xin Li, Ning Yi Jin, Guang Ze Zhu, Hui Jun Lu

**Affiliations:** ^1^ Academician Workstation, Changchun University of Chinese Medicine, Changchun, China; ^2^ College of Animal Science and Technology, Jilin Agricultural University, Changchun, China; ^3^ Changchun Veterinary Research Institute, Chinese Academy of Agricultural Sciences, Changchun, China; ^4^ Department of Specialty Medicine, School of Basic Medicine, Qingdao University, Qingdao, China

**Keywords:** norovirus, virus-like particles, macrophages, antigen presentation, phenotypic maturation, Th1

## Abstract

Norovirus (NoV) is a zoonotic virus that causes diarrhea in humans and animals. Outbreaks in nosocomial settings occur annually worldwide, endangering public health and causing serious social and economic burdens. The latter quarter of 2016 witnessed the emergence of the GII.P16-GII.2 recombinant norovirus throughout Asia. This genotype exhibits strong infectivity and replication characteristics, proposing its potential to initiate a pandemic. There is no vaccine against GII.P16-GII.2 recombinant norovirus, so it is necessary to design a preventive vaccine. In this study, GII.P16-GII.2 type norovirus virus-like particles (VLPs) were constructed using the baculovirus expression system and used to conduct immunizations in mice. After immunization of mice, mice were induced to produce memory T cells and specific antibodies, indicating that the VLPs induced specific cellular and humoral immune responses. Further experiments were then initiated to understand the underlying mechanisms involved in antigen presentation. Towards this, we established co-cultures between dendritic cells (DCs) or macrophages (Mø) and naïve CD4+T cells and simulated the antigen presentation process by incubation with VLPs. Thereafter, we detected changes in cell surface molecules, cytokines and related proteins. The results indicated that VLPs effectively promoted the phenotypic maturation of Mø but not DCs, as indicated by significant changes in the expression of MHC-II, costimulatory factors and related cytokines in Mø. Moreover, we found VLPs caused Mø to polarize to the M1 type and release inflammatory cytokines, thereby inducing naïve CD4+ T cells to perform Th1 immune responses. Therefore, this study reveals the mechanism of antigen presentation involving GII.P16-GII.2 recombinant norovirus VLPs, providing a theoretical basis for both understanding responses to norovirus infection as well as opportunities for vaccine development.

## Introduction

Norovirus (NoV) infections are a common cause of diarrhea outbreaks in humans and many animals. Presently, human-derived NoVs are one of the main pathogens involved in food-induced diarrhea, causing approximately 699 million infections (1–3) and 200,000 deaths worldwide each year (4). NoVs are extremely contagious, and even a few viral particles may cause infection (5). Epidemic episodes of NoV infection typically occur in semi-closed or closed environments, such as kindergartens, schools, nursing homes, hospitals, restaurants, cruise ships, or the military (2, 6).

At present, NoVs are principally classified into seven major genotypes (GI-GIV) based on gene sequences of the RNA-dependent-RNA-polymerase (RdRp) and major capsid proteins (VP1) (7). Of these, GI, most GII, along with a small number of GIV-type NoV can infect humans and cause epidemic acute gastroenteritis (AGE). Other NOV genotypes can infect cattle, pigs (8), dogs (9), cats, sheep and rodents. The currently known GI and GII-type NoV include no less than 31 genotypes (10). Since 2002, most major global epidemics associated with NoV have involved GII.4 (10, 11). However, in the fourth quarter of 2014 and 2015, a GII.17 type of NoV emerged in some Asian countries to become the main cause of diarrheal disease outbreaks (12–14). This highlighted the potential of non-GII.4 genotypes to become causes of major epidemics. Notably, in the winter of 2016, the number of norovirus outbreaks in China increased significantly compared with the previous four years. Of the 56 outbreaks in 2016, 79% of the outbreaks were caused by GII.P16-GII.2 recombinant NoV (7). GII.P16-GII.2 is a new type of Norovirus recombined by the RdRp gene of GII.P16 and the VP1 gene of GII.2 (15). Moreover, as shown by a recent study, the GII.P16-GII.2 recombinant NoV has the same replicability as the current pandemic GII.4 type, projecting the potential of GII.P16-GII.2 to cause new rounds of outbreaks and pandemic infections (15). GII.P16-GII.2 is extremely infectious to children (16). It can cause severe gastroenteritis and lead to adverse clinical outcomes. The results indicated that the first infection with GII.P16-GII.2 may cause a delay in virus clearance in most people (15).

Antigen presenting cells (APC) refer to a type of immune cells that ingest and process antigens which are then presented as processed antigens to T and B lymphocytes. Both DC and macrophages function as antigen-presenting cells (APC), which act as messengers between the innate and adaptive responses (APC to T cells) (17). Antigen capture serves to induce APC activation, inducing the expression of surface MHC molecules, costimulatory molecules (including CD80, CD86, CD40) and several related cytokines (18), allowing APC cells to effectively present antigens to T cells for antigen delivery. Macrophages are mainly divided into M1-type and M2-type (19). Previous reports (19) indicate the main functions of M1 macrophages include the mediation of pro-inflammatory responses, Th1 immune responses, antigen presentation, killing pathogens and inhibiting tumor formation. On the other hand, the functions of M2 macrophages include participation in tissue remodeling/reconstruction, mediating Th2-type immune regulation and angiogenesis.

Given the emerging significance of GII.P16-GII.2 recombinant NoV and its and potential to cause a pandemic, this study aimed to explore its immunological characteristics *in vivo* and *in vitro*. Towards this, we evaluated the immunogenic effects of VLPs of the GII.P16-GII.2 recombinant NoV in mice. We particularly focused on the effects of VLPs on DCs and macrophages in order to elucidate antigen presentation mechanisms. The findings presented here provide an increased understanding of the immune mechanisms involved, and moreover, establish the theoretical basis for the development of novel norovirus vaccines.

## Materials and Methods

### Plasmids and Cell Strains

The Bac-to-Bac Vector baculovirus expression system kit and DH10BacTM competent cells were purchased from Thermo Fisher. The VP1 gene of GII.P16-GII.2 Norovirus was cloned from strain Env/CHN/2016/GII.P16-GII.2/BJSMQ virus strain (GenBank accession number: KY421125.1). SF9 cells were purchased from BeNa Culture Collection (BNCC, China).

### Construction and Identification of Recombinant Baculovirus

The VP1 gene cloned from Norovirus GII.P16-GII.2 was integrated into the pFastBac-HTB vector using a seamless cloning kit. The recombinant plasmid (pFastBac-HTB-VP1) was then identified by PCR using pUC-M13F/pUC-M13R primers from the Bac-to-Bac Vector kit before constructing recombinant baculovirus according to the manufacturer’s instructions. SF9 cells were infected with norovirus recombinant baculovirus and were observed by western blot (WB), Indirect immunofluorescence analysis (IFA) and electron microscopy after infection.

### Purification of Virus-Like Particles

Suspensions of SF9 cells were infected with recombinant baculovirus. After culture for 5 days at 27°C with 115 rpm/min shaking, the cells were harvested, pelleted and disrupted by sonication. Crude protein extracts were prepared by centrifugation and the supernatants applied to an AKTA purification system to purify VLPs. The VLPs were then variously analyzed by SDS-PAGE, protein scanning, particle size analysis, and electron microscopic observation to evaluate the purification efficiency.

### Cellular Immune Response Detection

Six-week-old female C57BL/6 mice were randomly divided into two groups of 8 animals, one immunized with VLPs (VLP immunization group) and the other with PBS (Mock group). In this study, the BCA Protein Assay Kit (Beyotime Biotechnology, China) was used to determine the protein concentration of VLPs. The immunization group received an intramuscular injection of 50 ug VLPs at day 0 and a booster immunization at day 14 while the mock group received the same volume of PBS. On day 28, the mice were euthanized and splenic lymphocytes isolated using a lymphocyte separation kit (Tian Jin Hao Yang Biological Manufacture Co., Ltd, China), according to the manufacturers’ instructions. Thereafter, immunostaining and flow cytometry was used to analyze the expression of lymphocyte subsets. Total and activated B lymphocytes were detected using APC-CD19 and FITC-CD40, respectively; T lymphocyte subtypes were detected using PECy5-CD3, FITC-CD4 and PE-CD8; memory CD8+ T lymphocytes were detected using APC-CD3, FITC-CD8, PECy7-CD44 and PE-CD62L; CD8+ effector memory T cell (Tem) were defined as the CD3+CD8+CD44+CD62L- population while CD8+ central memory T cells (Tcm) were defined as the CD3+CD8+CD44+CD62L+ population. All flow cytometry antibodies used were purchased from Biolegend (San Diego, CA, USA). The T lymphocyte proliferation detection experiment was operated in accordance with the method reported in the previous literature (20).

### Specific Antibody Detection

Another new batch of C57BL/6 mice were divided into VLPs immunization group (8 mice) and two control groups (4 mice in each group) consisting of immunization with PBS alone or PBS and adjuvant. The adjuvant used in this experiment was alum(Thermo Fisher Scientific, catalog number 77161). The VLP immunization group was immunized with 50μg of purified VLPs and adjuvant, and the two control groups were adjuvant alone and PBS alone. All groups were injected intramuscularly. 14 days and 28 days after the first immunization, the booster immunization was performed in the same way. Blood was collected from the retroorbital socket every week, and the serum separated and stored. Specific antibody detection was carried out as previously described (21). Purified norovirus VP1 recombinant protein obtained by prokaryotic expression was used to coat ELISA plates at 2.5 μg/ml, and sample of mouse sera pre-diluted at 1:800 for analysis. At 8th week, dilute the sera gradually of VLPs group by 2 times for endpoint specific antibody titer detection, P/N>2.1 is regarded as positive.

### The Effect of VLPs on Phenotypic Maturation of Dendritic Cells and Macrophages

Six-week-old female C57BL/6 mice were purchased from Beijing Vital River Lab Animal Technology Co., Ltd. Dendritic cells (DCs) were isolated according to previously published methods (22). Macrophages were obtained from mouse bone marrow-derived cells. The cells were cultured in 1640 medium supplemented with 10 ng/mL M-CSF and 10% FBS in 6-well plates for 6 days with the medium changed every 2 days.

Isolated dendritic cells or macrophages were exposed to VLPs (10μg) or mock treated for 48 hours before assessing changes in the expression of cell surface molecules and their production of cytokines using flow cytometry and ELISA, respectively. The cells were stained with APC-CD11c, APC-F4/80, PE-CD80, FITC-CD86, FITC-MHC-II, and FITC-CD40 antibodies (all from Biolegend, San Diego, CA, USA) and changes in the percentage (%) of cells expressing these markers determined. The levels of cytokines (TNF-α, IL-6 and IL-12p70) were detected using cytokines ELISA kits purchased from R&D Systems (Minneapolis, MN, USA).

### The Influence of VLPs on Mø Polarization

Naive CD4+ T cells were isolated using the EasySep™ Mouse Naïve CD4+ T Cell Isolation Kit (STEMCELL Technologies). Mø cells and naïve CD4+T were mixed and incubated at a ratio of 1:5 for 3 hours, and then VLPs were added and incubated for 48 hours. After VLPs, Mø cells and naïve CD4+T cells were incubated for 48 hours and the cells subjected to flow cytometric analysis by staining with FITC-CD206, APC-CD11C and PE-F4/80 to determine the effects of VLPs on Mø polarization.

### Analysis of Antigen Presentation *In Vitro*

Mø cells and naïve CD4+T were mixed and incubated at a ratio of 1:5 for 3 hours, and then VLPs were added and incubated for 48 hours. After culture, the cells were collected and stained with APC-CD3 and FITC-CD4 antibodies. Thereafter, the cells were treated with Cytofix/Cytoperm Fixation/Permeabilization solution (BD, San Diego, CA, USA) and further stained with antibodies against cytokines (PE-IL-4 and PEcy7-IFN-γ) before flow cytometric analysis. The culture supernatants were also collected and the levels of inflammation-related cytokines (IL-6, TNF-α, IL-18) detected by ELISA. Parallel samples of cells were reserved for Western blotting assessment using the Reactive Inflammasome Antibody Sampler Kit (Cell Signaling Technology).

### Statistical Analysis

All experiments were performed independently at least thrice and results were presented as means ± standard deviation (SD). Student’s t tests were applied to compare the differences between two groups. One-way analysis of variance (ANOVA) was employed to determine significance of differences among multiple groups. Significance levels were defined as *p < 0.05, **p < 0.01, ***p < 0.001 and ****p < 0.0001.

### Ethical Statement

All animal experiments were performed according to the guidelines of the Animal Welfare and Research Ethics Committee of Changchun University of Chinese Medicine (Approval ID: 2020259).

## Results

### Identification and Purification of VLPs

**Figure 1:**
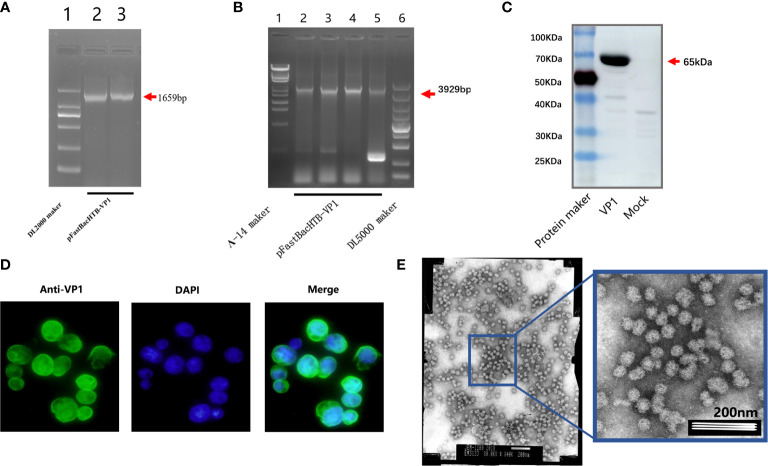
Identification of virus-like particles. PCR amplification of the recombinant plasmid pFastBac-HTB-VP1 using VP1 specific primers **(A)**. PCR amplification the pFastBac-HTB-VP1 showing a recombinant rod-shaped plasmid using pUC/M13F and pUC/M13R primers **(B)**. Western blot analysis detecting norovirus VP1 protein in SF9 cells infected with pFastBac-HTB-VP1 or a mock control **(C)**. The cells from **(C)** were analyzed by indirect immunofluorescence staining against VP1 (green) or after DAPI staining to reveal cell nuclei (blue) **(D)**. Electron microscopic observation of SF9 cells after recombinant baculovirus infection revealing abundant VLPs **(E)**.

The successful integration of the VP1 gene sequence into the pFastBac-HTB-VP1 plasmid was revealed by PCR analysis using VP1 specific primers (**Figure 1A**) along with the confirmation of the recombinant rod-shaped plasmid using amplification with the pUC/M13F and pUC/M13R primers (**Figure 1B**). Thereafter, SF9 cells were infected with the recombinant VP1 baculovirus or mock for 5 days before further analysis. Instructively, Western blot analysis of the infected cells using anti-NoV VP1 antibodies revealed a strong band at 65kDa, consistent with the size of the VP1 protein (**Figure 1C**) and moreover, indirect immunofluorescence analysis of the cells revealed strong fluorescence staining against VP1 in infected cells but not controls (**Figure 1D** and data not shown**)**. Finally, analysis of the electron microscopy revealed the abundance of similarly sized and shaped VLPs (**Figure 1E**). Together these results indicated that the recombinant baculovirus was successfully constructed and expressed with abundant expression of the recombinant VP1 protein detected in SF9 cells.

**Figure 2:**
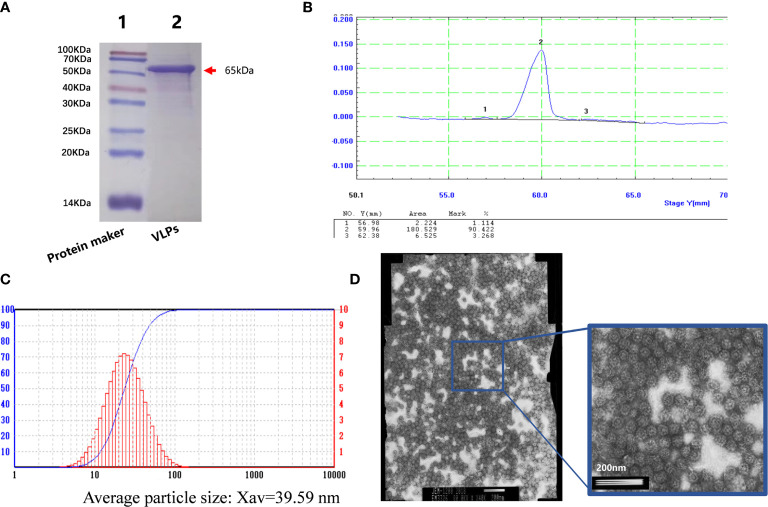
Purification of virus-like particles (VLPs). SDS-PAGE analysis of purified VLPs obtained from SF9 cells infected with the recombinant baculovirus for 5 days. Purification was performed using the AKTA purification system. **(A)**. Densitometric scanning profile of the SDS-PAGE results from **(A)** showing the purification efficiency was greater than 90% **(B)**. Particle size analysis of the purified VLPs **(C)**. Electron micrograph of the purified VLPs **(D)**.

After this confirmation, we prepared purified VLPs for use in the immunization experiments. Baculovirus infected SF9 cells were collected and disrupted by sonication before applying the crude protein extracts to an AKTA purification system. Thereafter, the purified VLPs were concentrated. Analysis of the samples by SDS-PAGE showed a single protein (**Figure 2A**) with densitometric analysis indicating that the purity of the VLP sample was greater than 90% (**Figure 2B**). We then subjected the purified VLPs to particle size analysis where it was found that the average particle size was 39.59nm, which was similar to the size of norovirus (**Figure 2C**). Lastly, the purified VLPs were observed under the electron microscope where highly uniform particles were seen (**Figure 2D**). Thus, together these findings indicated that the VLPs obtained were of high purity and suitable for use in subsequent experiments.

### VLPs Can Induce Cellular Immune Responses

**Figure 3:**
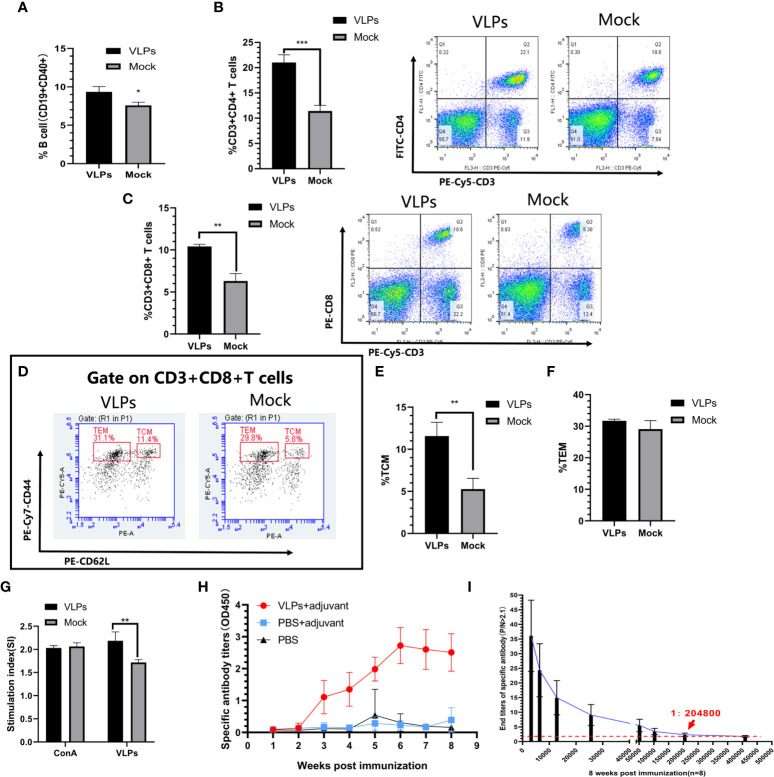
Evaluation of immune responses in VLP-immunized mice. Splenic lymphocytes were isolated from VLP or Mock immunized mice on the 28th day after the first immunization and analyzed by flow cytometry. B cell activation was measured as the percentage of CD19+CD40+ cells **(A)**. Percentage of CD3+CD4+ T cells **(B)** and CD3+CD8+T cells **(C)**. Gating strategy to detect CD8+ central memory T cells (TCM: CD3+CD8+CD44+CD62L+) and CD8+ effector memory T cells (TEM: CD3+CD8+CD44+CD62L-) **(D)**. Percentages of TCM **(E)** and TEM **(F)** calculated from **(D)**. T lymphocyte proliferation detection **(G)**. Weekly analysis of norovirus-specific antibodies in serum after another new batch of mice was immunized with purified VLPs and adjuvant, PBS and adjuvant or PBS alone **(H)**. Titration analysis of norovirus-specific antibody levels in sera from the VLP-immunized mice collected at 8 weeks post-immunization **(I)**. All experiments were performed independently at least thrice and results were presented as means ± standard deviation. Significance levels were defined as *p < 0.05, **p < 0.01 and ***p < 0.001.

To assess cellular immune responses to VLPs, splenic lymphocytes were isolated on day 28 from mice receiving an initial immunization on day 0 and booster immunization on day 14. Using flow cytometry, we first compared the activation efficiency of B cells in the VLP immunization and Mock groups as the ratio of CD40+CD19+ lymphocytes. We observed that the ratio of CD40+CD19+ cells in the VLP-immunized animals was higher than for the controls indicating that VLPs could activate B cells (**Figure 3A**). In parallel, we also analyzed T lymphocyte subtypes using flow cytometry to measure the relative expression of CD3, CD4 and CD8 markers. This analysis revealed that the proportion of CD3+CD4+ and CD3+CD8+ positive T cells was significantly increased in the VLP immunization group compared to the Mock group, indicating that VLPs can effectively induce cellular immune responses (**Figures 3B, C**).

CD8+ T cells can directly eliminate pathogens to provide host defense while CD8+T memory cells can provide enhanced protective immunity against re-infection (23, 24). Further flow cytometric analyses to discriminate memory CD8+ T lymphocyte populations indicated that immunization with VLPs resulted in significant increases in CD8+ central memory T cells (Tcm)compared to the Mock group (p<0.01) along with a non-significant trend of higher levels of CD8+ effector memory T cells (Tem) (p>0.05) (**Figures 3D–F**). Together this shows that VLPs could induce production of memory CD8+ T lymphocytes.

Following secondary infection with a pathogen, T lymphocytes rapidly proliferate to clear the infection. Towards assessing this function, we compared the ability of T lymphocytes to proliferate in response to re-stimulation with VLPs. Notably, the proliferation of T lymphocytes in the VLP group was significantly higher than for the Mock group (p<0.01) (**Figure 3G**). This implies that the VLP immunized mice possess the capacity to mount a rapid cellular immune response involving T-cells against infections with NoV.

In summary, these experiments established that immunization with VLPs induced a cellular immune response.

### VLPs Induces High Levels of VP1-Specific Antibodies

Another new batch of mice was immunized for specific antibody detection. As an extension to the previous section which suggested that humoral immunity was invoked by VLP immunizations, we measured the levels and persistence of VP1-specific antibodies in mice over an 8-week period. After immunization, serum levels of VP1-specific antibodies began to be detected after 3 weeks and plateaued at 6 weeks whereas no specific antibodies could be detected in the Mock immunized animals (**Figure 3H**). Furthermore, comparing the serum antibodies titers in the VLP immunization group at the 8th week we found the titers reached 1:204800 (**Figure 3I**). Thus, immunization with purified VLPs resulted in the specific and effective production of norovirus-specific antibodies.

### VLPs Cannot Promote the Maturation of DCs

**Figure 4:**
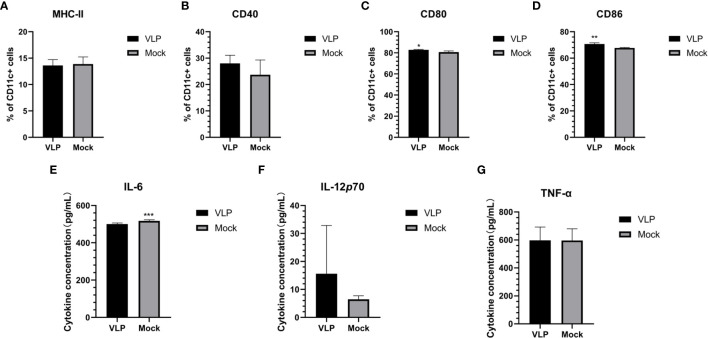
Analysis of DC maturation in response to VLPs. Cultured DCs isolated from murine bone marrow were treated with PBS (Mock) or VLPs (10μg) for 48 h respectively. Thereafter, flow cytometry was used to compare the percentages of CD11c+ DCs expressing MHC-II **(A)**, CD40 **(B)**, CD80 **(C)** and CD86 **(D)**. In parallel, culture supernatants were harvested to measure the secreted levels of IL-6 **(E)**, IL-12p70 **(F)** and TNF-α **(G)**. All experiments were performed independently at least thrice and results were presented as means ± standard deviation. Significance levels were defined as *p < 0.05, **p < 0.01 and ***p < 0.001.

To test if the VLPs could function to promote the maturation of DCs, we incubated with isolated DCs with or without VLPs. After 48 hours, we used flow cytometry to assess key phenotypic changes in the cells along with subjecting the supernatants to relevant cytokine analyses. However, we found the cell surface expression levels of MHC-II and CD40 on the DCs were not significantly different between the VLP and Mock treated groups (**Figures 4A, B**). Nonetheless, the CD80 (p<0.05) and CD86 (p<0.01) surface expression of DCs was significantly higher in the VLP group compared to the Mock group (**Figures 4C, D**). Moreover, ELISA tests for cytokines revealed that the expression of IL-6 was significantly lower in the VLP group (p<0.001, **Figure 4E**) whereas there was a non-significant trend that both IL-12P70 and TNF-α levels were higher in VLP *versus* control group comparisons (**Figures 4F, G**). The results indicate that while VLPs can be recognized by DC cells, they do not effectively induce DC maturation.

### VLPs Effectively Activate Macrophage

**Figure 5:**
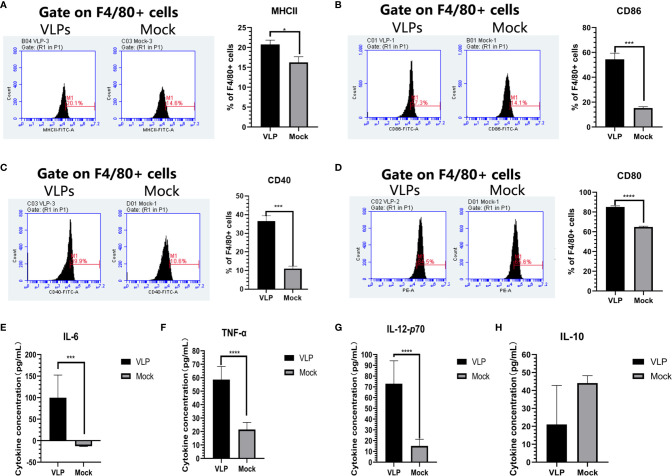
Analysis of macrophage activation in response to VLPs. Cultured macrophage isolated from murine bone marrow were treated with PBS (Mock) or VLPs (10μg) for 48 h respectively. Thereafter, flow cytometry was used to compare the percentages of F4/80+ macrophage expressing MHC-II **(A)**, CD80 **(B)**, CD40 **(C)** and CD86 **(D)**. In parallel, culture supernatants were harvested to measure the secreted levels of IL-6 **(E)**, TNF-α **(F),** IL-12p70 **(G)** and IL-10 **(H)**. All experiments were performed independently at least thrice and results were presented as means ± standard deviation. Significance levels were defined as *p < 0.05, ***p < 0.001 and ****p < 0.0001.

We next sought to establish if VLPs can elicit macrophage activation by incubating isolated Mø cells with VLPs. After 48 hours, the cells and culture supernatant were collected for flow cytometry and cytokine analysis, respectively. Flow cytometric analysis indicated that surface MHC-II expression of Mø was significantly increased in the VLP group relative to the Mock group (p<0.05, **Figure 5A**). Similarly, the cell surface expression of CD40 and CD86 molecules on Mø were also significantly higher in the VLP group (p<0.001, **Figures 5B, C**). Moreover, CD80 expression was also significantly higher in the VLP group (p<0.0001, **Figure 5D**. Similarly, the results of ELISA assays showed that IL-6 (p<0.001), IL-12P70 (p<0.0001) and TNF-α (p<0.0001) were all significantly higher in the VLP group (**Figures 5E–G**) while the expression of IL-10 was higher in the Mock group (**Figure 5H**). Since activated Mø are known to highly express surface molecules such as MHC-II, CD40, CD80 and CD86, and also secrete IL-6, IL-12P70 and TNF-α cytokines to promote antigen presentation, these findings propose that the norovirus VLPs effectively activate macrophage.

### Macrophages Can Recognize VLPs for Antigen Presentation

As independent verification that Mø can effectively recognize VLPs, we studied the ability of Mø to undergo chemotaxis towards VLP in Transwell^®^ migration assays. Isolated Mø were added to upper chambers while the lower chambers were filled with 600 ul culture medium with (VLPs) or without VLPs (Mock). After 24 hours, the Transwell inserts were removed and stained with crystal violet to estimate the number of migratory cells. This analysis revealed more Mø migrated towards VLPs compared to the Mock group (**Figure S1A**), indicating that macrophages can effectively recognize VLPs for antigen presentation.

A second but important step involved assessing whether the APCs (DC or Mø) were capable of presenting VLP-derived antigens to T cells. Towards this, we isolated naïve CD4+ T cells using the EasySep™ Mouse Naïve CD4+ T Cell Isolation Kit. Preliminary experiments indicated the purity of both CD4+ T cells and naïve CD4+ T populations was above 98% (**Figures S2A, B**). Thereafter, DCs (**Figure S2C**) or Mø (**Figure S2D**) was incubated with naive CD4+T cells at a ratio of 1:5 for 3 hours before the addition of VLPs for a further 48 hours. The cells were then collected for flow cytometric analysis of CD4 in combination with fixation and intracellular detection of the IL-4 and IFN-γ cytokines. The results showed that the positive rates of CD4+IL-4+ and CD4+IFN-γ+ cells in Mø co-cultures were higher than for the DCs co-cultures, and the ratio of CD3+CD4+IFN-γ+ cells was significantly higher than that in the DC group (p< 0.0001, **Figure S1B**). These experiments established that the antigen presentation capabilities of Mø are more effective than DCs in presenting VLP-derived antigens to naive CD4+ T cells.

### VLPs Promote Mø to M1-Type Polarization for Antigen Presentation

**Figure 6:**
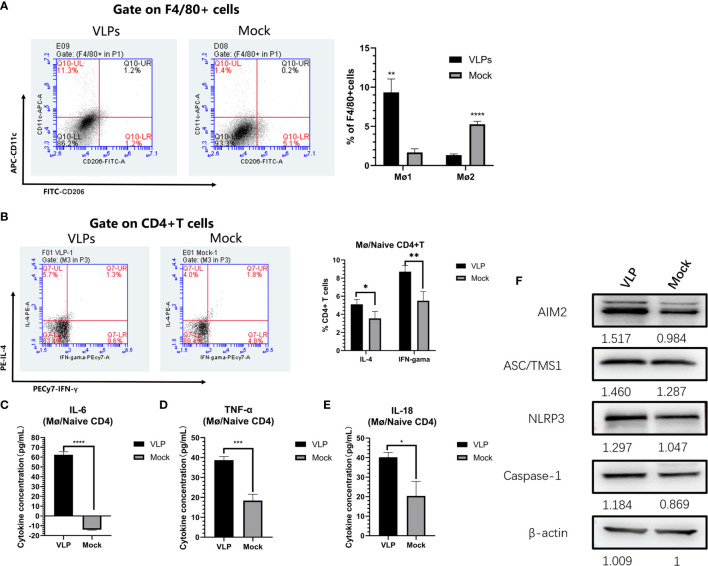
*In vitro* analysis of antigen presentation in macrophages. Macrophages and naïve CD4+T were mixed and incubated at a ratio of 1:5 for 3 hours, before treatment with PBS (Mock) or VLPs (10μg) for 48 h, respectively. Thereafter, flow cytometry was used to compare macrophage polarization **(A)** and the cellular expression of IL-4 and IFN-γ in CD4+ T cells **(B)**. In parallel, culture supernatants were harvested to measure the secreted levels of IL-6 **(C)**, TNF-α **(D)** and IL-18 **(E)**. The co-cultured cells were harvested and key proteins in the NLRP3 inflammation pathway measured by Western blotting **(F)**. All experiments were performed independently at least thrice and results were presented as means ± standard deviation. Significance levels were defined as *p < 0.05, **p < 0.01, ***p < 0.001 and ****p < 0.0001.

We next used the Mø-naïve CD4+T cell co-culture model to assess and effects of VLPs on Mø polarization. As before, Mø was incubated with naive CD4+T cells at a ratio of 1:5 for 3 hours before the addition of VLPs for a further 48 hours. Thereafter, the cells were stained with FITC-CD 206, APC-CD11c and PE-F4/80 and analyzed by flow cytometry. The results showed that the proportion of M1-type (F4/80+CD11c+CD206-) cells in the VLP group was significantly increased compared to the Mock group (p<0.01), while conversely the proportion of M2-type (F4/80+CD11c-CD206+) cells was decreased in the VLP group (p<0.0001). This suggests that VLPs can induce Mø polarization from the M2-type towards the M1-type (**Figure 6A**).

Parallel measurements of the levels of IL-4 and IFN-γ expression in the CD4+ T cells showed that the ratios of CD4+IL-4+ (p<0.05) and CD4+IFN-γ+ (p<0.01) T cells in VLP group were significantly higher than those in the Mock group (p<0.01) (**Figure 6B**). This indicates that Mø can present norovirus antigens to naive CD4+ T cells, inducing Th0 cells to differentiate into Th1 and Th2 cells, but with Mø more inclined to induce Th1 immune responses. Furthermore, assessment of inflammatory cytokine levels in the culture supernatants showed that IL-6, TNF-α, and IL-18, were all significantly higher in the VLP group compared to the Mock group (p<0.0001, **Figure 6C**; p<0.05, **Figure 6D** and p<0.001, **Figure 6E** respectively). These data suggest that VLPs activate Mø for antigen presentation through inflammatory pathways.

Finally, in order to further analyze the activation mechanisms in Mø, we used Western blotting to detect changes in inflammation-related proteins in the cells collected from the co-culture model. This analysis showed that the expression levels of key protein in the NLRP3 inflammation pathway, namely AIM2, ASC/TMS1 and NLRP3, were all increased in VLP group cultures (**Figure 6F**), indicating that VLPs may activate Mø for antigen presentation through the NLRP3 pathway.

## Discussion

Virus like particles represent a very effective platform for antiviral immunity research, with beneficial properties including good immunogenicity, low allergenicity and high clinical efficacy. Presently, the application of specific VLPs has been proven effective in preventing viral-related diseases. For example, vaccinations with VLPs incorporating antigens from papilloma virus and hepatitis B virus amongst others have been shown to be well tolerated and effective at inducing immunity in the clinical setting (25–27). In this study, a baculovirus expression system was used to successfully obtain GII.P16-GII.2 recombinant norovirus VLPs similar in shape and in size to the natural norovirus. Moreover, particle size analysis indicated the average VLP size was 39.59nm, similar to the size previously reported for norovirus (28).

CD8+ T cells provide host defense and protective immunity by directly binding and eliminating foreign pathogens. Immature CD8+ T cells from secondary lymphoid organs enter the peripheral blood, look for “non-self” antigens and activate proliferation and differentiation into effector cells to kill pathogens. Eventually, the remaining effector cells are then transformed into long-term CD8+ T memory cells, which can provide enhanced protective immunity against re-infection (23, 24). In this study, we demonstrated that immunization of mice with the VLPs effectively induced cellular immune responses involving the production of memory CD8 + T cells (**Figures 3D–F**). VLP immunization also effectively activated B cells (**Figure 3A**) and stimulated the production of norovirus specific antibodies. Antibody levels plateaued at 5 weeks and 8 weeks after immunization the titre of norovirus VP1-specific antibodies reached 1:204800 (**Figure 3I**). These overall findings indicate that the GII.P16-GII.2 recombinant norovirus VLPs shows excellent immunogenicity with resulting cellular and humoral immune responses, proposing the VLPs as a strong candidate for vaccine development.

The immunogenicity of vaccines mainly depends on antigen recognition and presentation by APCs. After recognition, vaccine antigens are internalized through phagocytosis and subsequently broken down into peptide fragments that are re-presented to naïve CD4+ T cells. Thus, the key point of any immunization strategy is to ensure the vaccine is effectively recognized and ingested by the APCs, especially macrophages (Mø) and dendritic cells (DC) (27). We therefore, explored the ability of professional APCs (Mø and DCs) to recognize and respond to the GII.P16-GII.2 recombinant norovirus VLPs. Importantly we found that the norovirus VLPs did not effectively induce DC maturation and antigen presentation since the expression of surface MHC-II molecules on the DCs did not significantly change (**Figure 4**). However, we found the norovirus VLPs could effectively activate Mø. Exposure of Mø to VLPs resulted in high surface expression of MHC-II and the co-stimulatory factors CD40, CD80 and CD86, these being key indicators of activation and antigen presentation (**Figures 5A–D**). Moreover, we found that VLPs also increased the production of pro-inflammatory cytokines (IL-6, IL-12p70 and TNF-α) in Mø (**Figures 5E–G**), providing further indications that VLPs can activate Mø for antigen presentation. Finally, we used migration experiments to further explore the ability of Mø to recognize VLPs. Notably, Mø showed chemotaxis towards VLPs, indicating they were capable of effectively recognizing and responding to VLPs. Thus, we found Mø but not DCs were effectively activated by norovirus VLPs, providing important insights into the effective application of VLPs in vaccination strategies.

To further explore the ability of macrophages to recognize and process VLP antigens, we turned to a co-culture system to simulate interactions between Mø and naive CD4+ T cells. Exposure of the co-cultures to VLPs resulted in increases in the proportion of M1-type macrophages, indicating that VLPs promote polarization towards the M1 phenotype. As established in previous studies, the M1-type macrophage has the function of mediating antigen presentation. Moreover, previous reports have shown that IL-10 mediates the M2-type polarization of Mø (19). Consistent with the ability of VLPs to induce M1-type polarization, we found that incubation of Mø with VLPs resulted in the decreased expression of IL-10 in culture supernatants (**Figure 5H**). Collectively these experiments proposed that the norovirus VLPs were internalized by Mø and processed for antigen presentation. We then further complemented these studies by investigating the phenotype of T cells in the co-culture model.

First, we explored the effects of norovirus VLP-stimulated macrophages on the differentiation of naïve CD4+ T cells, using the relative expression of the cytokines IL-4 and IFN-γ in CD4+ T cells. The results of this experiment showed that the expression of IL-4 and IFN-γ in CD4+ T cells were significantly increased (**Figure 6B**), indicating that Mø can present VLP antigens to naïve CD4+ T cells, and induce the differentiation of Th0 cells towards Th1 and Th2 phenotypes. However, Th1 cells have a higher degree of differentiation, which indicates that VLPs are more inclined to cellular immune responses. Furthermore, consistent with our results, previous studies have revealed that M1-type macrophages have the function of mediating Th1 immune responses (19). We also tested the culture supernatants for the levels of inflammatory cytokines and found that IL-18, IL-6 and TNF-α were all increased in co-cultures exposed to VLPs (**Figures 6C–E**), indicating that Mø antigen presentation may be activated through inflammatory pathways. Following this lead, we then examined the expression of key NLRP3 inflammation pathway proteins. Indeed, the expression levels of AIM2, ASC/TMS1 and NLRP3 were all increased in co-cultures treated with VLPs (**Figure 6F**), indicating that VLPs may activate macrophage for antigen presentation through the NLRP3 pathway.

This study has some limitations. Firstly, we explored the mechanisms underlying the effects of VLPs on macrophages and antigen presentation through *in vitro* experiments, but not *in vivo*. There is a lack of correlation between *in vitro* and *in vivo* studies on this mechanism, which may introduce bias. Secondly, we did not conduct challenge experiments, mainly due to the difficulties in long-term culturing of viruses and the lack of animal models, which altogether hinder the identification of protection relevance (29). The research group is also actively looking for an effective human norovirus (HuNoV) infection model to facilitate the evaluation of norovirus vaccine candidates. At present, murine norovirus (MNV) has proven invaluable as a tool to dissect host and viral factors that contribute to viral persistence, as well as to assess the critical role of host factors in regulating intestinal infections (29–31). An important difference in genome organization between HuNoV and MNV is the presence of a fourth overlapping reading frame (ORF4) in ORF2, which is unique to MNV encoding virulence factor 1 (VF1) (29, 32). However, MNV model has obvious limitations as an alternative method of HuNoV infection (32). For example, MNV-infected mice did not show symptoms such as diarrhea and vomiting. Moreover, MNV is a chronic infection model, while HuNoV is mainly an acute infection (33, 34). Despite the above-mentioned limitations, MNV is still the preferred model for studying the pathogenesis of norovirus. Therefore, follow-up studies will try to evaluate candidate vaccines using the MNV model, so as to address one of the limitations of this study.

In summary, we investigated the potential of VLPs expressing VP1 from the GII.P16-GII.2 recombinant norovirus as a vaccine candidate in mice. We established evidence for resulting humoral and cellular immunity and explored the underlying mechanisms. We found that macrophage but not DC can be activated by VLPs and that the activated macrophages process VLP antigens and present them to naive CD4+ T cells for promoting Th1 immune responses. Along with revealing these mechanistic aspects, this study provides a theoretical basis for developing VLP-based vaccines against norovirus.

## Data Availability Statement

The original contributions presented in the study are included in the article/**Supplementary Material**. Further inquiries can be directed to the corresponding authors.

## Ethics Statement

All animal experiments were performed according to the guidelines of the Animal Welfare and Research Ethics Committee of Changchun University of Chinese Medicine (Approval ID: 2020259).

## Author Contributions

NJ, HL, GZ, and JH were responsible for experiment design and drafting the manuscript. JH, QL, JF, JZ, QL, SL, CC, CX, FN, HZ, and ZL performed experiments and analyzed data. All authors have read and approved the final manuscript.

## Funding

This work was supported by National Science and Technology Major Project of the Ministry of Science and Technology of China [No. 2018ZX10102-001] and the Young Scientist Program Training Program of Changchun University of Traditional Chinese Medicine [QNKXJ2-2021ZR31].

## Conflict of Interest

The authors declare that the research was conducted in the absence of any commercial or financial relationships that could be construed as a potential conflict of interest.

## Publisher’s Note

All claims expressed in this article are solely those of the authors and do not necessarily represent those of their affiliated organizations, or those of the publisher, the editors and the reviewers. Any product that may be evaluated in this article, or claim that may be made by its manufacturer, is not guaranteed or endorsed by the publisher.
