# Supplementary material for: GII.P16-GII.2 Recombinant Norovirus VLPs Polarize Macrophages Into the M1 Phenotype for Th1 Immune Responses
Source: Front Immunol. 2021 Nov 18;12:781718. doi: 10.3389/fimmu.2021.781718 (PMC8637406; doi:10.3389/fimmu.2021.781718)
Supplement: Supplementary file 1 [file DataSheet_1.docx]

1. **Supplementary materials and methods**

1.1 Transwell migration assay

Transwell plates (6.5 mm Transwell® with 8.0 µm pore polycarbonate membranes, Corning) were used to measure the chemotaxis of macrophages towards VLPs over 24 hours. Macrophages (5×10^5^ cells) are added to the upper chamber of Transwell, and the lower chamber is filled with 600 ul containing VLPs (10μg). In the mock group, the same volume of PBS was added to the lower chamber as a control. After 24 hours of incubation, the migration number of macrophages was analyzed by crystal violet color.

1. **Supplementary figure legends**

**Supplementary Fig.S1 Recognition and antigen presentation of VLPs by macrophages.** Microscopic images of crystal violet stained Transwell membranes comparing the chemotaxis of macrophages towards media with VLPs or without VLPs (Mock) **(A)**. Macrophages or dendritic cells were mixed with CD4+ T cells and the co-cultures further incubated with VLPs for 48 hours. Flow cytometry was then used to assess the intracellular expression of IL-4 and IFN-γ in CD4+ T cells **(B)**. All experiments were performed independently at least thrice and results were presented as means ± standard deviation. Significance levels were defined as *p < 0.05, **p < 0.01, ***p < 0.001 and ****p < 0.0001.

**Supplementary Fig.S2 Verification of naïve CD4+ T cells isolation and co-culture models of macrophages or dendritic cells with naïve CD4+ T cells.** Naïve CD4+T cells were isolated using the EasySep™ Mouse Naïve CD4+ T Cell Isolation Kit. The enrichment of CD4+ and naïve CD4+ T cells before and after sorting was assessed using flow cytometry **(A)** with the results recorded as percentages of total cells **(B)**. Microscopic observation of dendritic cells **(C)** or macrophages **(D)** co-cultured with naïve CD4 +T cells. The red and green arrows indicate dendritic cells and macrophages, respectively, while the blue arrows show naïve CD4+T cells. All experiments were performed independently at least thrice and results were presented as means ± standard deviation. Significance levels were defined as *p < 0.05, **p < 0.01, ***p < 0.001 and ****p < 0.0001.
