# Supplementary figures and images for: GII.P16-GII.2 Recombinant Norovirus VLPs Polarize Macrophages Into the M1 Phenotype for Th1 Immune Responses
Source: Front Immunol. 2021 Nov 18;12:781718. doi: 10.3389/fimmu.2021.781718 (PMC8637406; doi:10.3389/fimmu.2021.781718)

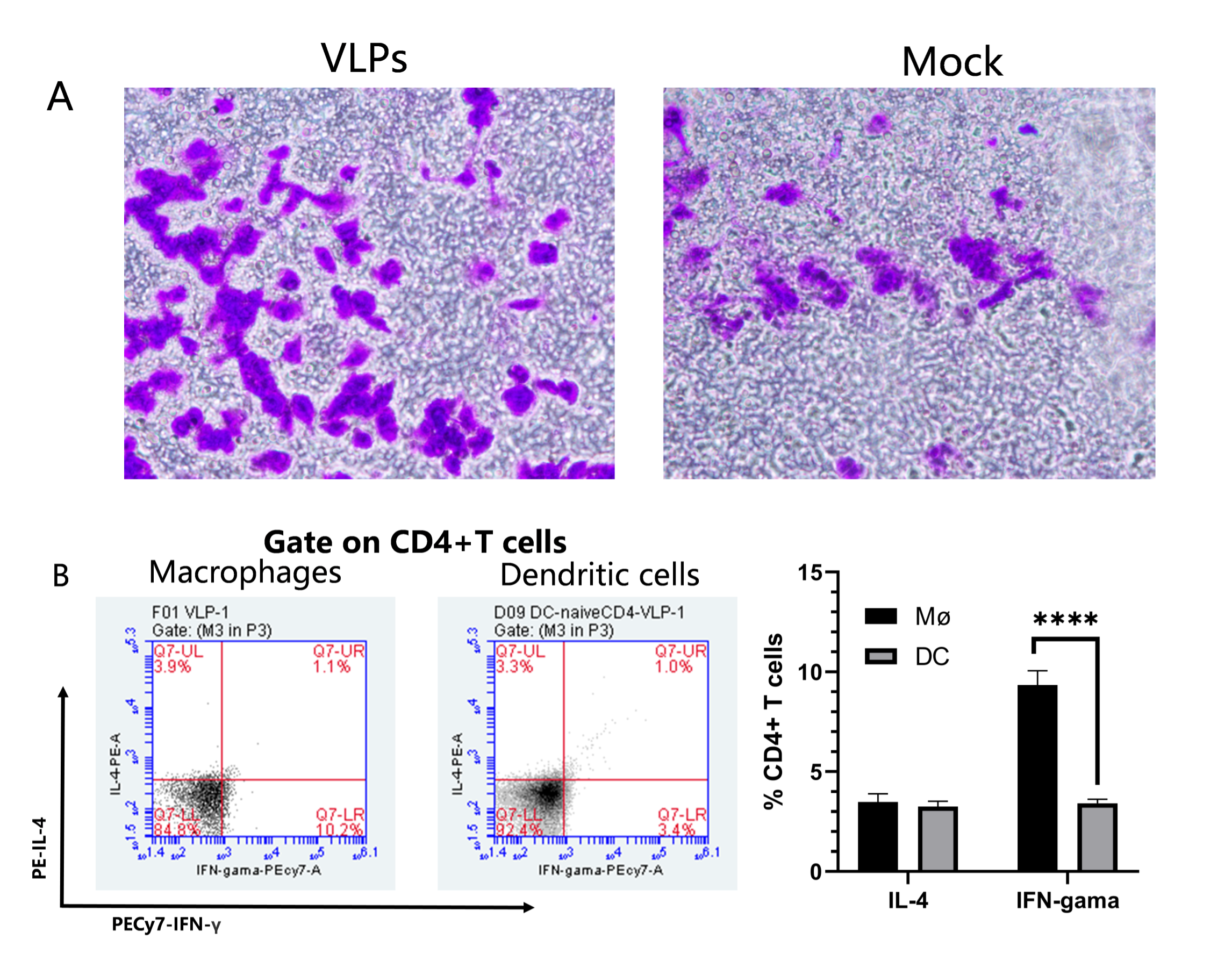

Supplement: Supplementary file 2 [file Image_1.tif]

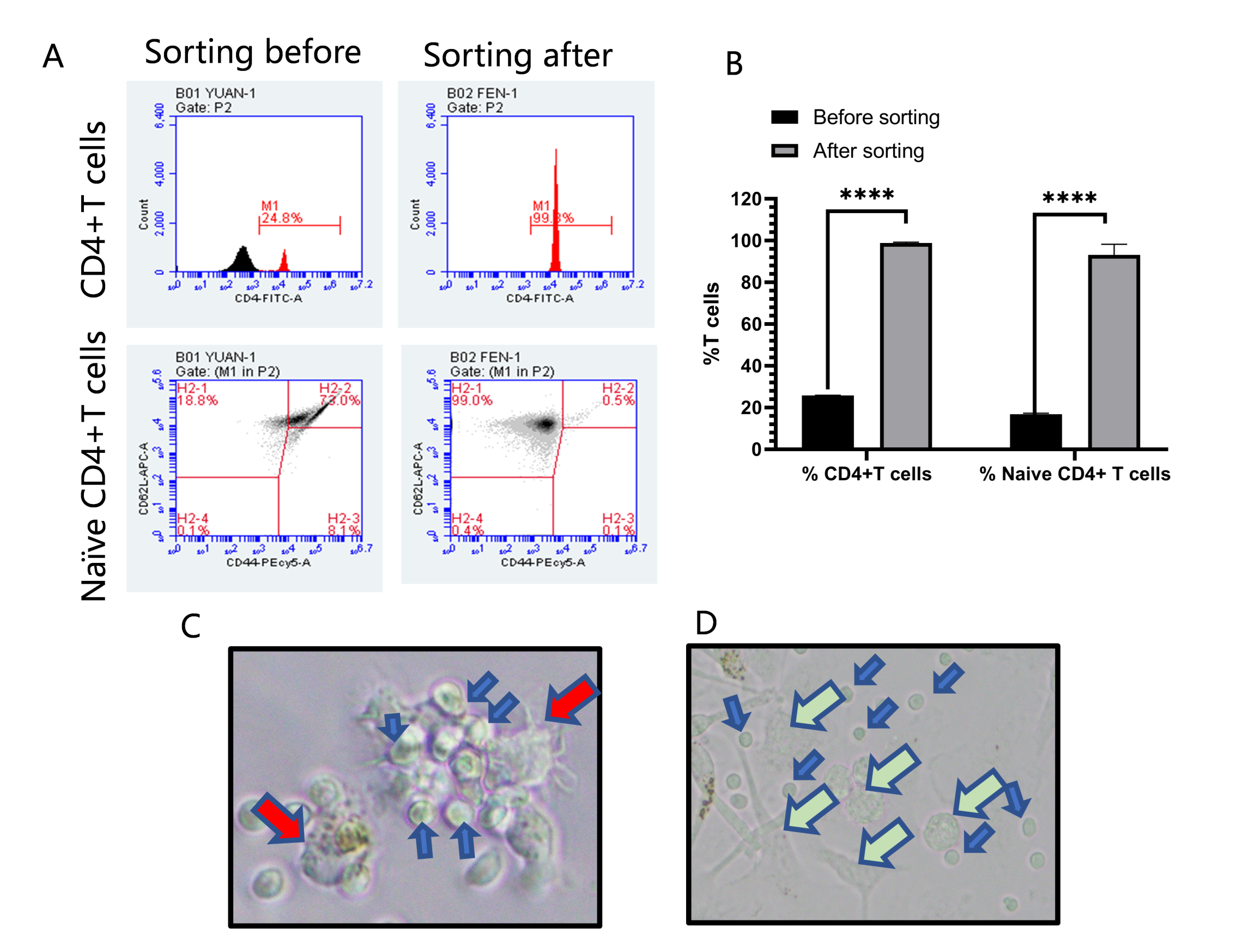

Supplement: Supplementary file 3 [file Image_2.tif]
